# Effects of transcranial direct current stimulation combined with Bosu ball training on the injury potential during drop landing in people with chronic ankle instability

**DOI:** 10.3389/fphys.2024.1451556

**Published:** 2024-08-14

**Authors:** Xueke Huang, He Gao, Haitao Fu

**Affiliations:** ^1^ Graduate school, Shandong Sport University, Jinan, China; ^2^ School of physical education, Shandong Sport University, Jinan, China

**Keywords:** chronic ankle instability, landing, ankle sprain, transcranial direct current stimulation, Bosu ball training

## Abstract

**Purpose:**

To investigate the effects of transcranial direct current stimulation (tDCS) combined with Bosu ball training on the injury potential during drop landing in people with chronic ankle instability (CAI).

**Methods:**

A total of 40 participants with CAI were recruited and randomly divided into the tDCS + Bosu and Bosu groups. The people in the tDCS + Bosu group received intervention of tDCS combined with Bosu ball training, and those in the Bosu group received intervention of sham tDCS and Bosu ball training, for 6 weeks with three 20-min sessions per week. Before (week_0_) and after (week_7_) the intervention, all participants drop-landed on a trap-door device, with their affected limbs on a moveable platform, which could be flipped 24° inward and 15° forward to mimic an ankle inversion condition. The kinematic data were captured using a twelve-camera motion capture system. Two-way ANOVA with repeated measures was used to analyze data.

**Results:**

Significant group-by-intervention interactions were detected in the peak ankle inversion angular velocity (*p* = 0.047, η^2^_p_ = 0.118), the time to peak ankle inversion (*p* = 0.030, η^2^_p_ = 0.139), and the plantarflexion angle at the moment of peak ankle inversion (*p* = 0.014, η^2^_p_ = 0.173). Post hoc comparisons showed that compared with week_0_, the peak ankle inversion angular velocity and the plantarflexion angle at the moment of peak ankle inversion were reduced, the time to peak ankle inversion was advanced in both groups at week_7_, and the changes were greater in the tDCS + Bosu group compared to the Bosu group. And, a significant intervention main effect was detected in the peak ankle inversion angle in the two groups (*p* < 0.001, η^2^_p_ = 0.337).

**Conclusion:**

Compared with the Bosu ball training, the tDCS combined with Bosu ball training was more effective in reducing the injury potential during drop landing in people with CAI.

## 1 Introduction

Ankle sprains are one of the most common sports injuries (Fong et al., 2007), accounting for approximately 40% of all sports injuries (Colville, 1998), with recurrence rates as high as 70%–80% (McKay et al., 2001). Approximately 40% of people experienced ankle sprains developed to chronic ankle instability (CAI) (Doherty et al., 2016; Hertel and Corbett, 2019). Those with CAI are characterized by recurrent ankle sprains with persistent symptoms such as pain, recurrent episodes, swelling, limited motion, weakness, and self-reported functional impairment (Hertel and Corbett, 2019). The recurrent sprains make people with CAI prone to experience long-term degenerative sequelae such as post-traumatic ankle osteoarthritis (Hintermann et al., 2002), reduced physical activity levels (Hubbard-Turner and Turner, 2015), and decreased health-related quality of life (Arnold et al., 2011). In the United States, approximately 2 million people suffer from acute ankle sprains each year (Feger et al., 2017), with a medical cost of about $6.2 billion (Gribble et al., 2016).

Drop landing is a common maneuver in sports activities requiring strong dynamic stability and a common scenario for lateral ankle sprains (LAS) due to excessive ankle inversion (Doherty et al., 2014). Ankle sprains occur mostly on the lateral side at a rate of up to 90% (McKay et al., 2001), and approximately 75% of LAS occur during landing (Taghavi Asl et al., 2022). Ankle joints are easy to be inverted and/or plantarflexed during landing (Li et al., 2019; Kim et al., 2021; Stotz et al., 2021), and arthrogenic muscle inhibition of the peroneal muscles is observed in people with CAI, which can lead to decreased peroneal muscles strength (Dong et al., 2024), and ankle sprains are prone to occur when the peroneal muscles fail to resist ankle inversion in time (Jeon et al., 2021).

Ankle inversion angle and angular velocity, time to peak ankle inversion, and plantarflexion angle at the moment of peak ankle inversion are key indicators of the ankle sprain at landing. The peak ankle inversion angle (Simpson et al., 2022) and peak ankle inversion angular velocity (Terrier et al., 2014) were greater in people with CAI compared to those without CAI. When the ankle is accidently inverted, the distance between the talus and fibula increases (Fong et al., 2012), and the ligaments connecting the bones are stretched, which increases the potential of ligament injury (Medina McKeon and Hoch, 2019; Huang et al., 2021). Compared to those without CAI, those with CAI reached peak ankle inversion later after landing, during which time the ankle was in a state of instability and the foot was unable to adjust to the proper position to better absorb the ground reaction forces during landing (Terada and Gribble, 2015; Delahunt and Remus, 2019), resulting in the transfer of excessive ground reaction force to the joint surfaces and surrounding ligaments, increasing the potential of ankle injury and ultimately causing ankle sprain (Terada and Gribble, 2015; Delahunt and Remus, 2019). Excessive inversion and plantarflexion of the ankle during landing are the main biomechanical reasons responsible for ankle injuries (Caulfield et al., 2004). Compared to people without CAI, those with CAI landed with a greater plantarflexion angle (Delahunt et al., 2007), which increases stretching of the lateral ankle ligaments (Wright et al., 2013).

Many conventional interventions for CAI are symptom-driven, meaning that they are designed to rehabilitate deficits caused by CAI, such as strength and sensory deficits (McKeon and Donovan, 2019). However, such interventions were less effective, and people continued to experience ankle instability or experience re-injury after those interventions (McKeon and Wikstrom, 2016; Wright et al., 2017). It has been pointed out that physiologic changes in the mechanoreceptors of injured ankle ligaments or muscles are not the only factors for functional abnormalities, the maladaptive neuroplastic changes in the central nervous system (CNS), especially in the cerebral cortex, also affects the recovery of ankle function (Riemann, 2002). The adaption of CNS induces impaired sensorimotor and neurocognitive function (Needle et al., 2017; Maricot et al., 2023), and the cortical excitability decreased in the primary motor cortex (M1) in people with CAI (Needle et al., 2017; Bruce et al., 2020). In challenging condition, the inactivation of M1 leads to insufficient cognitive resources, causing abnormal movement patterns and injuries (Burcal et al., 2019; Bruce et al., 2020).

Transcranial direct current stimulation (tDCS), a noninvasive neuromodulation technique that modulates excitability and promotes neuroplasticity in the target cortex (Geiger et al., 2017), may be one of the options for CNS rehabilitation in people with CAI. tDCS has been proven to improve the excitability of the M1 and muscle activation in people with CAI (Bruce et al., 2020). Another key consideration in the use of tDCS is the selection of the motor task with which it is paired (Stagg and Nitsche, 2011), as one of its primary uses is as an adjunctive therapy to augment the acquisition of the task, i.e., motor learning. Bosu ball training can be one of the options to pair with tDCS for two reasons. Bosu balls have uneven surfaces on which instability training can be performed, and instability training has been shown to be effective for balance and postural control in people with CAI (Ha et al., 2018); And, during Bosu ball practice, participants may perform successive movements to counteract perturbations of the center of gravity, in which the CNS may learn skills continuously to counteract perturbations, and tDCS facilitates this learning process.

Therefore, this study aimed to investigate the effects of tDCS combined with Bosu ball training on the injury potential during drop landing in people with CAI, by comparing the effects of Bosu ball training only. It is hypothesized that 1. both tDCS combined with Bosu ball training and the Bosu ball training would decrease injury potential during drop landing in people with CAI, represented by the reduced peak ankle inversion angle, peak ankle inversion angular velocity, the plantarflexion angle at the moment of peak ankle inversion, and the advancement of the time to peak ankle inversion. And 2, the tDCS combined with Bosu ball training would have better effectiveness than the Bosu ball training only.

## 2 Materials and methods

### 2.1 Sample size estimate

An *a priori* power analysis (G*Power Version 3.1) indicated that a minimum of 22 participants are needed to obtain an alpha level of 0.05 and a statistical power of 0.80 based on a previous report, which compared the peak ankle inversion angular velocity in people with and without CAI when performing inversion landings under anticipated and unanticipated conditions (η^2^_p_ = 0.064) (Han et al., 2023).

### 2.2 Participants

Seventy-five participants were assessed for eligibility, and 40 of them were recruited by distributing flyers at local universities. Following the guidelines of the International Ankle Consortium (Gribble et al., 2013), the inclusion and exclusion criteria were set as follows.

Inclusion criteria: Participants must have experienced at least one severe ankle sprain a year prior, causing pain, swelling, or inflammation that prevented normal participation in daily activities for more than 1 day; have had at least two episodes of ankle ‘giving way’ in the past 6 months; feel a persistent sense of ankle instability and functional impairment in daily activities; and score less than 24 on the Cumberland Ankle Instability Tool (Hiller et al., 2006).

Exclusion criteria: Participants with a history of lower-extremity fracture or who had undergone surgery in the past year; those who had experienced acute injuries such as lower-extremity sprains within the past 3 months; those with bilateral CAI.

The 40 participants were randomly divided into tDCS + Bosu and Bosu groups. All participants signed their approved written informed consent forms before participation. Human participation was approved by the Ethics Committee of Exercise Science of Shandong Sport University (2022043) and was in accordance with the Declaration of Helsinki.

### 2.3 Study design

In this single-blinded and sham-controlled study. Forty participants were randomly assigned to either the tDCS + Bosu group or the Bosu group in a 1:1 ratio. The randomization was done using a web-based randomization service (www.randomization.com). Details of the assigned group were written on cards and concealed using sequentially numbered opaque sealed envelopes. Participants in the tDCS + Bosu group underwent tDCS combined with Bosu ball training, and participants in the Bosu group underwent sham tDCS and Bosu ball training, for 6 weeks with three 20-min sessions per week. Injury potential was measured before and after the 6-week intervention.

### 2.4 Bosu ball training

**FIGURE 1:**
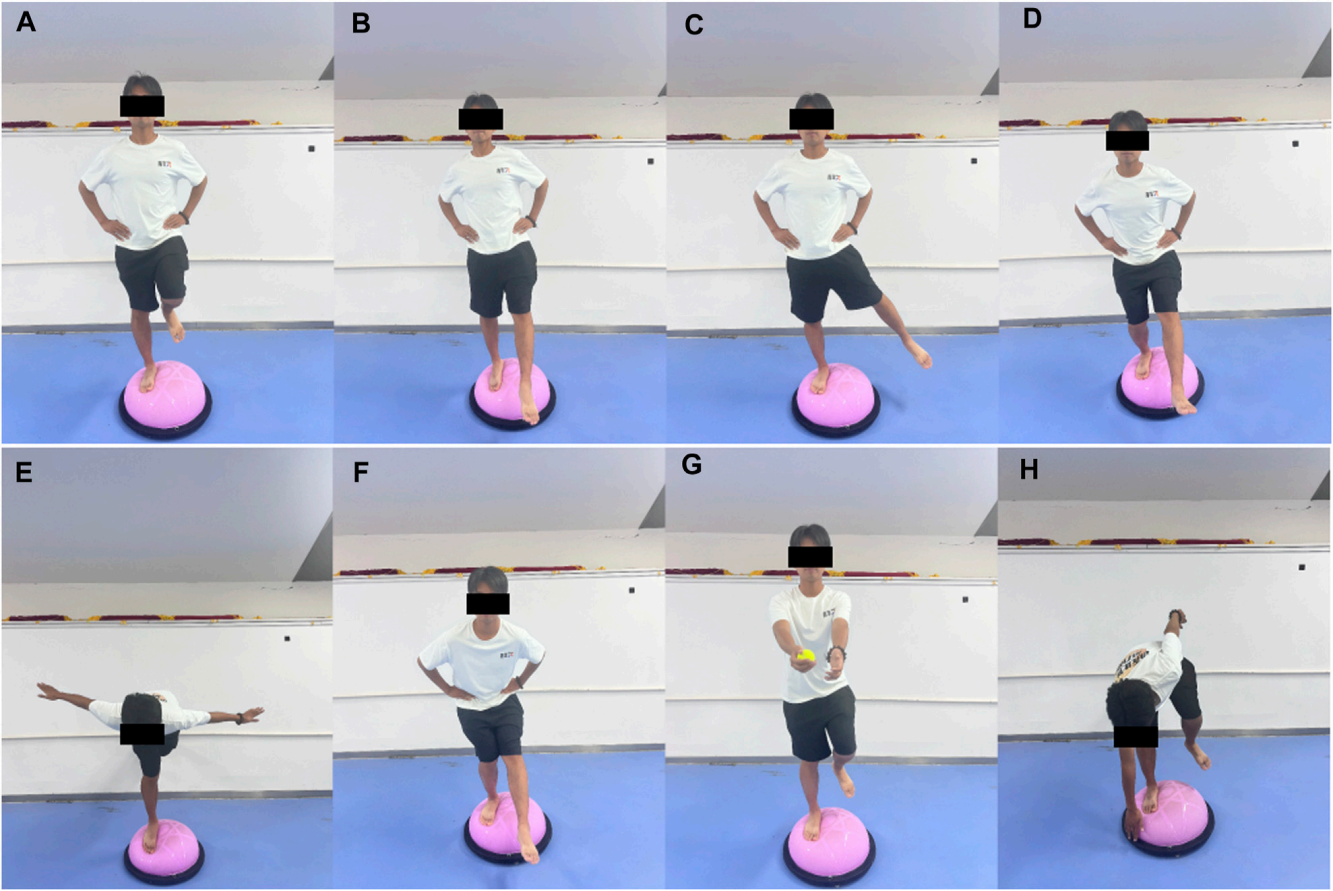
Illustrations of the Bosu ball training movements **(A)** single-leg stance, **(B)** single-leg stance with forward-backward leg swing, **(C)** single-leg stance with medial-lateral leg swing, **(D)** single-leg squat, **(E)** swallow balanced stance, **(F)** single-legged squat take-ups, **(G)** catching a ball while single-leg stance, and **(H)** bending over to touch the edge while single-leg stance.

Participants perform Bosu ball training barefoot, using a progressive training program. In weeks 1 and 2, they practiced single-leg stance, single-leg stance with forward-backward leg swing (30°–45°), single-leg stance with medial-lateral leg swing (20°–30°), and single-leg squat. In weeks 3 and 4, they practiced swallow balanced stance, single-leg stance with forward-backward leg swing (45°–60°), single leg stance with medial-lateral leg swing (30°–45°), and single-legged squat take-ups. In weeks 5 and 6, they practiced catching a ball while single-leg stance, single-leg stance with forward-backward leg swing (45°–60°), single-leg stance with medial-lateral leg swing (30°–45°), and bending over to touch the edge while single-leg stance. See illustrations of the movements in Figure 1. Each movement was performed for 30 s and repeated 5 times, with a 30-s rest between movements. The total time of each session is approximately 20 min.

### 2.5 tDCS intervention

**FIGURE 2:**
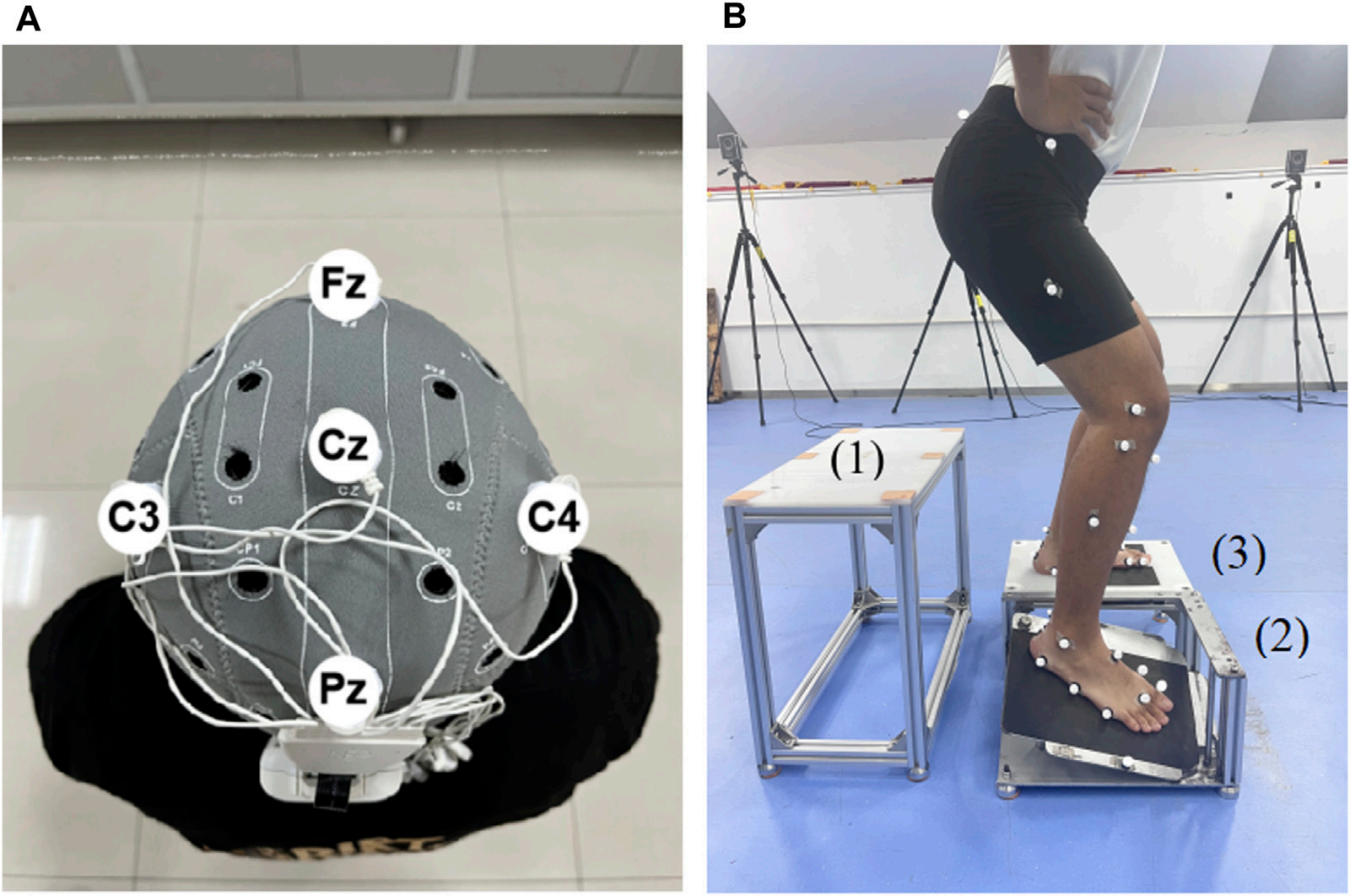
Illustration of tDCS electrode placement and drop landing test **(A)** The illustration of the tDCS electrode placement. The anode was placed over Cz of the 10/20 EEG template; the four cathodes were placed over Fz, C3, Pz, and C4. **(B)** The illustration of the drop landing test. 1, take-off platform. 2, moveable platform. 3, support platform.

tDCS was delivered by a tDCS device (StarStim8, NE transcranial direct current stimulator, Spain). Five 5 mm radius rubberized circular electrodes were used in the tDCS montage, with a central anodal electrode and four surrounding cathodal (return) electrodes. According to the 10/20 EEG template, the anode was placed at Cz, and the other four electrodes were located at Fz, Pz, C3 and C4, respectively (Villamar et al., 2013) (Figure 2A). The current intensity at the anode was set at 2 mA, and the return current was evenly distributed among the four cathodes. During tDCS, the current increased from 0 mA to 2 mA in the first 30 s, was maintained at this level for 19 min, and then gradually decreased to 0 mA in 30 s. For the sham stimulation, the same electrode location was used, but the current remained to be 0 mA during the stimulation.

### 2.6 Drop landing test

Before formal tests, participants wore uniform tight shorts and T-shirts. Then 36 markers were adhered to their lower limbs according to the protocol of the Oxford Foot Model (McCahill et al., 2008). Participants then had 5 min to warm up and familiarize themselves with the tests by at least 5 drop-landing trials. Then, they conducted formal drop landing test.

Participants drop-landed from a height of 30 cm (Mokhtarzadeh et al., 2017) onto a custom trap-door device consisting of three platforms, namely, take-off (Figure 2B), movable (Figure 2B), and support (Figure 2B) platforms. The device is commonly used to trigger ankle inversion during landing (Gehring et al., 2014). The surface of the movable platform would be flipped 24° inward and 15° forward when it suffered a force greater than 10 N. Each participant’s affected foot landed on the movable platform and the unaffected foot landed on the support platform. A marker was placed on the lateral edge of the movable platform to identify the time point when the platform surface moved. During the drop-landing test, participants’ kinematic data were recorded using a 12-camera, 3D infrared motion capture system (Vicon Vantage V5, Oxford Metrics Limited, Oxford, United Kingdom) at a frequency of 100 Hz. Participants wore a jacket with a rope that passed through a pulley on the ceiling, with the other end of the rope controlled by the tester for safety. A total of five trials were performed, and means of the five trials were calculated for further analysis.

### 2.7 Data processing

The data were collected from the time point at landing and 200 ms after landing (Simpson et al., 2022), which was defined by the movement of the marker placed on the lateral edge of the movable platform (Bhaskaran et al., 2015) (Figure 2B). The time stage was selected because real ankle sprain occurs within this stage (Fong et al., 2009). The raw data from the motion capture system were processed using Vicon Nexus (version: 2.10.2, Oxford Metrics, Ltd.), imported into Visual 3D software (V6 Professional, C-Motion, United States) and low-pass filtered at 10 Hz (Jackson, 1979). High-frequency data, usually caused by alternating current or ground vibration, have been filtered.

### 2.8 Variables

The peak ankle inversion angle was defined as the maximum Euler angle of the foot relative to the tibia in the coronal plane. The peak ankle inversion angular velocity was defined as the maximum rate of changes of the ankle inversion angle, i.e., the maximum value of angular increment per unit time. The time to peak ankle inversion was defined as the time from the foot contact with the moveable platform to peak ankle inversion. And, the plantarflexion angle at the moment of peak ankle inversion was defined as the Euler angle between the line from the heel to the third metatarsal head and the tibia in the sagittal plane, at the moment of peak ankle inversion.

### 2.9 Statistical analysis

The normality of data was verified using Shapiro-Wilk tests. A two-way ANOVA with repeated measures was used to verify the main effects of group (tDCS + Bosu vs. Bosu) and intervention (week_0_ vs. week_7_), and their interactions. If a significant interaction was detected, Bonferroni adjusted post hocs would be conducted. Partial eta square (η^2^_p_) was used to represent the effect size of main effects and interactions. The thresholds for η^2^_p_ were as follows: 0.01–0.06, small; 0.06–0.14, moderate; >0.14, large (Pierce CA and Aguinis, 2004). Cohen’s *d* was used to represent the effect size of the *post hoc* comparison. The thresholds for Cohen’s *d* were as follows: <0.20, trivial; 0.21–0.50, small; 0.51–0.80, medium; >0.81, large (Cohen, 2013). All data are expressed as mean ± standard deviation. The significance level is set to 0.05 or 5%, and *p*-value less than the level indicates a statistically significant result, meaning the observed data provide strong evidence against the null hypothesis.

## 3 Results

**FIGURE 3:**
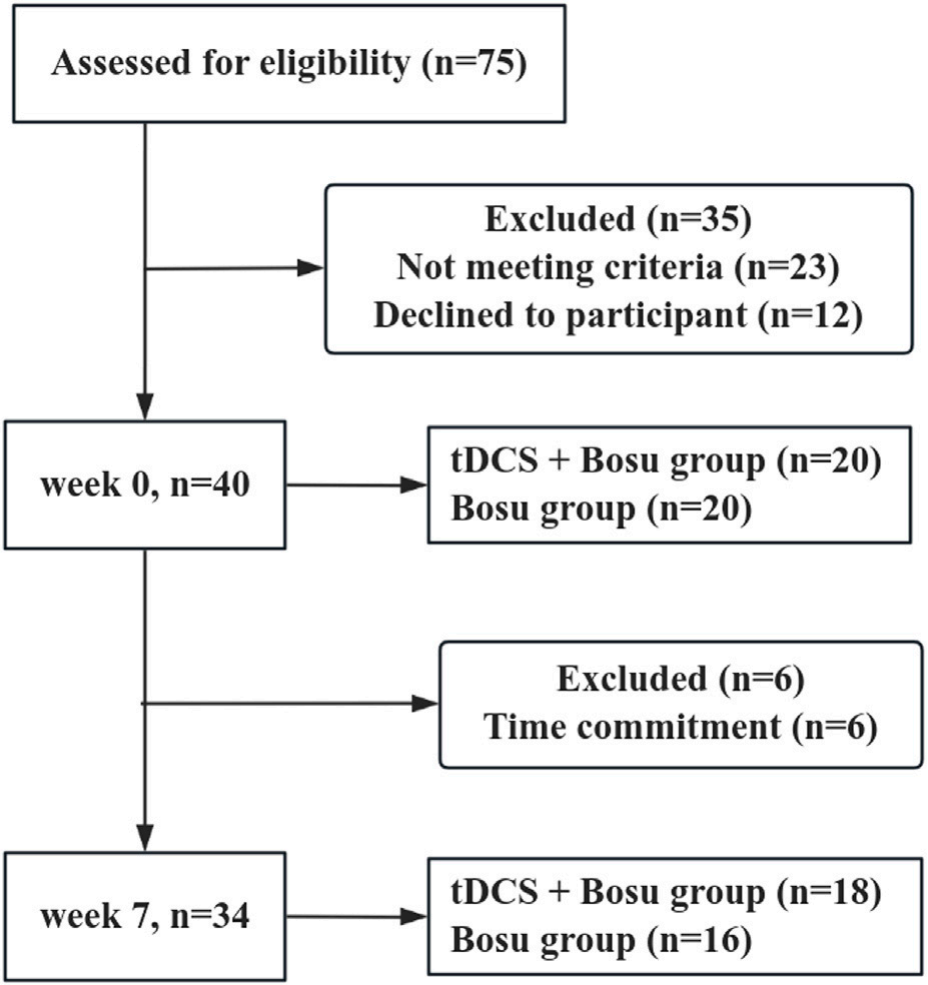
Participant flow chart. Participation flow chart from week 0 to week 7. The final analysis included data from 34 participants. Forty-one participants were excluded from the original 75 assessed due to various reasons.

The Shapiro-Wilk test showed that all dependent variables were normally distributed. Figure 3 shows that a total of 40 participants were recruited for this experiment and were divided into two groups. During the intervention, six participants were excluded due to time commitment, and after the 6-week intervention, 18 participants remained in the tDCS + Bosu group (20.1 ± 1.3 years; 175.5 ± 8.0 cm; 72.4 ± 9.6 kg) and 16 in the Bosu group (21.0 ± 1.8 years; 173.3 ± 12.0 cm; 68.9 ± 11.5 kg). There were no significant differences in age, height, and body mass between the two groups.

**FIGURE 4:**
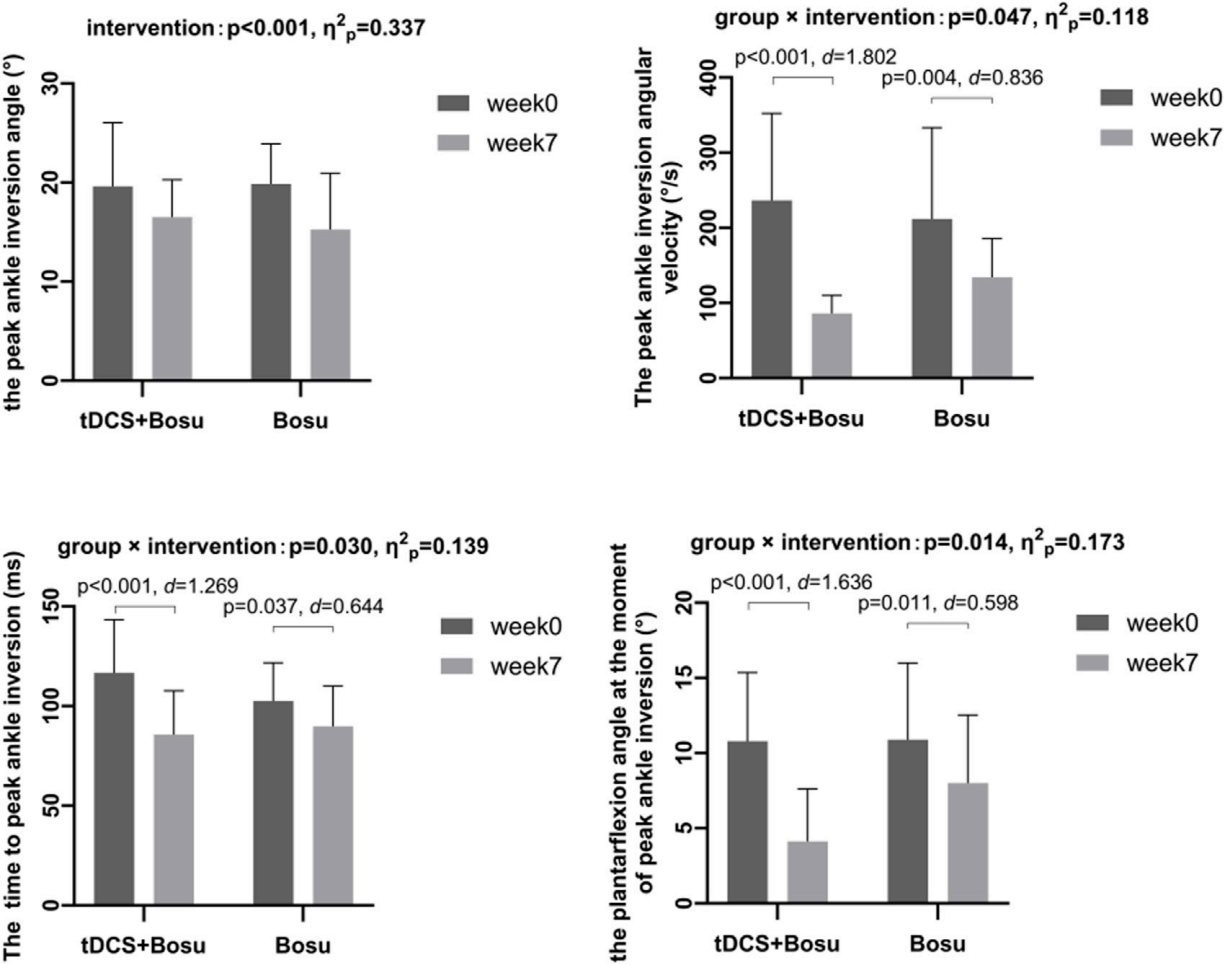
Comparison of kinematic data during drop-landing before and after interventions in people with chronic ankle instability *p < 0.05 indicates a statistical difference.

Figure 4 shows significant group by intervention interactions in peak ankle inversion angular velocity (*p* = 0.047, η^2^_p_ = 0.118), time to peak ankle inversion (*p* = 0.030, η^2^_p_ = 0.139), and plantarflexion angle at the moment of peak ankle inversion (*p* = 0.014, η^2^_p_ = 0.173). After 6 weeks of intervention, the ankle peak inversion angular velocity and the plantarflexion angle at the moment of peak ankle inversion were reduced in both groups, and the time to peak ankle inversion was advanced in both groups, and the changes were greater in the tDCS + Bosu group compared to the Bosu group. Moreover, a significant intervention main effect in both groups was detected in the peak ankle inversion angle (*p* < 0.001, η^2^_p_ = 0.337).

## 4 Discussion

The purpose of this study was to verify the effect of tDCS combined with Bosu ball training on the injury potential during drop landing in people with CAI. These results partially support our hypotheses by pointing out that tDCS + Bosu training has better effects in reducing peak ankle inversion angular velocity and the plantarflexion angle at the moment of peak ankle inversion and advancing time to peak ankle inversion than Bosu training only, and both two interventions reduced the peak ankle inversion angle.

Our results showed that Bosu ball training as well as tDCS + Bosu ball training reduced the peak ankle inversion angle, and tDCS + Bosu ball training did not show an extra effect. It could be inferred that most of the effects were due to Bosu ball training. We believe that the effectiveness of Bosu ball training lines in the role of the unstable surface and its potential to improve balance and postural control. Previous studies support us by pointing out that compared to a stable surface, training on an unstable surface has a better effect on balance and postural control in people with CAI (Ha et al., 2018). The use of unstable surface disrupts balance, increases the sensory stimulation needed between the joints and the skin, and enhances the body’s balance response (Alizamani et al., 2023). Furthermore, training on an unstable surface is believed to improve proprioception (Cug et al., 2016; Alizamani et al., 2023), which has been a deficit in people with CAI and can lead to a decrease in their postural control (Freeman et al., 1965; Liu et al., 2024). We believe that Bosu ball training improves balance and proprioception, so the participants could feel the changes in ankle movements earlier, and be able to limit the ankle inversion angle during the drop landing to decrease injury potential. The inability of tDCS to further improve proprioception may be related to the underlying pathomechanism of CAI (Ma et al., 2020). The diminished proprioception in people with CAI due in part to diminished spinal cord-mediated sensory afferent input from mechanoreceptors and/or diminished postural reflex responses (Lephart et al., 1998). tDCS selectively modulates cortical excitability and thus may have limited effects on the spinal control component of the proprioceptive system (Ma et al., 2020). In addition, it has been shown that while the application of tDCS over the affected motor cortex contributes to the recovery of lower limb motor weakness, this improvement is not sufficient to restore balance function (Chang et al., 2015).

Our study showed that tDCS + Bosu ball training had better effects than Bosu ball training in reducing injury potential during drop landing in people with CAI, which may be attributed to three reasons. Primary, tDCS may increase the activation of the peroneus longus and tibialis anterior, improve motor function, and reduce the injury potential in people with CAI. Studies have shown that increasing central nervous excitability improves muscle activation (Needle et al., 2017). It has been further pointed out that tDCS on M1 increased activation of peroneus longus (Bruce et al., 2020) and tibialis anterior (Zhan et al., 2023) in people with CAI. The peroneus longus and tibialis anterior are the main ankle eversion and dorsiflexion muscles, respectively, and increased activation of them reduces the ankle inversion and plantarflexion (Kim et al., 2003) as well as the inversion and plantarflexion moments during landing in patients with CAI (Li et al., 2018), which in turn reduces the ankle inversion angular velocity and plantarflexion angle, advances the time to peak ankle inversion, and thus reduces the potential for ankle injury.

Secondary, tDCS may improve the excitability of the M1 projected to lower limb muscles. It is believed that the M1, projected to peroneus longus and tibialis anterior, is smaller in size and less excitable (Pietrosimone and Gribble, 2012; Nanbancha et al., 2019) in people with CAI compared to those without CAI. A previous study pointed out that after tDCS intervention, stroke patients showed shorter latencies and higher motor evoked potential amplitudes in the tibialis anterior, compared to those receiving sham tDCS, suggesting that tDCS increases the excitability of corticospinal tract projected to the tibialis anterior (Chang et al., 2015). Another study further indicated that tDCS promoted M1 excitability and further enhanced the function of the muscles to which it projects (Sanes and Donoghue, 2000). In our study, tDCS may have facilitated the excitability of the M1 and further enhanced the function of peroneus longus and tibialis anterior, resisted the inversion and plantarflexion during trap-door drop landing, and then reduced the ankle sprain injury potential.

Tertiary, tDCS may enhance the effects of Bosu ball training by facilitating motor learning. Recent paradigm shifts in the etiology of ankle instability have revealed changes within the CNS that alter motor planning and produce movement patterns that re-injure individuals (Needle et al., 2017). A crucial consideration when using tDCS is to select a motor task to pair it with, as its primary purpose is to enhance the acquisition of a task as adjuvant therapy (Stagg and Nitsche, 2011). Previous studies supported our viewpoint by indicating that tDCS on M1 combined with the sequential sensorimotor task of sport stacking could better improve the flexibility of hands and motor performance compared to sham tDCS in healthy people (Pixa et al., 2017). It has also been shown that tDCS improved the accuracy of tracking during movements and led to greater improvements in ankle voluntary control of their paretic ankle in stroke patients than sham tDCS when practicing a tracking task for dorsiflexion-plantarflexion movements of the paralyzed ankle (Madhavan et al., 2011). We suggest that during Bosu ball training, people with CAI continually make movements to counteract perturbations of the center of gravity. During this process, the CNS continually learns the skills of counteracting perturbations, and tDCS facilitates this learning process.

To our knowledge, this study is the first to investigate the effect of tDCS combined with Bosu Ball training on the injury potential in people with CAI. It confirms that CNS-directed rehabilitation (e.g., tDCS) is an approach that can provide additional effects to conventional functional training for the prevention of ankle re-sprains in people with CAI, and provides new ideas for the clinical development of rehabilitation programs for people with CAI.

There are several limitations to this study. First, there was no follow-up after the 6-week intervention, so we were unable to determine how long the effect of the intervention on reducing the injury potential in people with CAI lasted. Second, the Bosu ball training was combined with either tDCS or sham tDCS, the individual effects of each intervention remain unknown. Third, we used four biomechanical variables to represent injury potential, however, no epidemiological studies explored the sensitivity and specificity of biomechanical predictors during drop landing to the ankle sprain occurrence. The use of statistical predictors would improve the precision of this study.

## 5 Conclusion

Compared with the Bosu ball intervention, the tDCS combined with Bosu ball intervention was more effective in reducing the injury potential during drop landing in people with CAI, suggesting that tDCS can be used as an effective rehabilitation approach to reduce the injury potential of ankle sprains in people with CAI.

## Data Availability

The original contributions presented in the study are included in the article/supplementary material, further inquiries can be directed to the corresponding author.
